# Morphological and Molecular Aspects of *In Vitro* Culture of Preantral Follicles Derived from Vitrified Ovarian Tissues Using A Two-Step Culture

**DOI:** 10.22074/cellj.2017.4264

**Published:** 2017-08-19

**Authors:** Mahboobeh Amoushahi, Mojdeh Salehnia, Seyed Javad Mowla, Nassim Ghorbanmehr

**Affiliations:** 1 Department of Anatomy, Faculty of Medical Sciences, Tarbiat Modares University, Tehran, Iran; 2 Department of Biotechnology, Faculty of Biological Sciences, Tarbiat Modares University, Tehran, Iran; 3 Department of Biotechnology, Faculty of Biological Sciences, Alzahra University, Tehran, Iran

**Keywords:** Vitrification, Folliculogenesis, Gene Expression, Sodium Alginate

## Abstract

**Objective:**

This study aimed to evaluate the expression of the genes related to folliculo-genesis after vitrification of mouse ovarian tissues using a two-step *in vitro* culture.

**Materials and Methods:**

In this experimental study, vitrified and non-vitrified ovaries from 7- day old (neonate) female mice were cultured using alpha-Minimum Essential Medium (α-MEM) supplemented with 5% fetal bovine serum (FBS) for 7 days. Morphology, surface area of ovaries and percentage of normal follicles were evaluated and compared in both groups. After one-week culture, in non-vitrified group, preantral follicles of cultured ovaries were isolated and cultured in a three-dimensional alginate culture system for 12 days. Then, the collected metaphase (M) II oocytes were inseminated with capacitated spermatozoa derived from 7-8-week old (adult) male NMRI mice. Follicular diameter, oocyte maturation, fertilization, embryo development and the expression of genes related to follicular development (*Pcna*, *Fshr* and *Cyp17a1*,) using real time reverse transcription-polymerase chain reaction (RT-PCR) were assessed at the end of last culture period in both groups.

**Results:**

The ovarian area in vitrified group (162468.20 703.78) was less than non-vitrified group (297211.40 6671.71), while the percentage of preantral follicles in vitrified group (18.40%) was significantly lower than those of non-vitrified group (24.50%) on day 7 of culture (P<0.05). There were no significant differences between the two groups in terms of follicular diameter, expression of genes related to development of follicles, oocyte maturation, fertilization, as well as embryo development (P>0.05).

**Conclusion:**

The results of this study showed that vitrification of ovarian tissue following *in vitro* culture had negative impact on the survival and development of follicles within the tissue. However, no significant alterations were observed in development, gene expression and hormonal production of *in vitro* culture of isolated follicles derived from vitrified ovarian tissues as compared to the non-vitrified samples.

## Introduction

Ovarian tissue cryopreservation is a good alternative method for preserving fertility in women suffering from premature ovarian failure (POF) or cancer that requires chemotherapy or radiotherapy (1). One method for the ovarian tissue cryopreservation of mammals, which has been widely studied, is vitrification (2). This method consists of a physical procedure, in which a high concentrated cryoprotectants applied without formation of any ice crystals to vitrified the living cells (3). Several studies have been conducted to improve ovarian vitrification method (4-7), while the successful results have been obtained on cow (8), mice (9, 10), rat (11) and human (12).

One of the methods to preserve ovarian fertility after cryopreservation is *in vitro* maturation of ovarian follicles, which has been applied in the recent investigations (13-15). Their results are different, but impact of vitrification on ovarian tissue is still ambiguous (16-18). It has been suggested that vitrification affects follicular development, leads to DNA damage and causes loss of some cytoplasmic mRNA (19). Moreover, a group of studies have reported some controversial results regarding the effects of vitrification methods on gene expression patterns of follicles in mammals (20-22). Fatehi et al. (23) have showed that the expression of some genes related to folliculogenesis (*Bmp15, Gdf9, BmprII, Alk6, Alk5, Has2,* and *Ptgs2*) had no effects on vitrified follicles cultured in a two-dimensional system as compared with the fresh control group. Asadzadeh et al. (24) have recently reported that expression of Timp-2 and Mmp-2 genes were altered after *in vitro* culture of isolated follicles derived from vitrified mouse ovarian tissue. Sampaio da Silva et al. (25) have recently showed that the proliferation of granulosa cells in developing follicles decreased after vitrification of ovarian tissue by changing in the gene expression of Cx43 in secondary follicles.

Therefore, study of the pattern of follicular gene expression in short- and long-term culture may be a useful method to assess the impact of vitrification on the development and maturation of follicles and oocytes. A number of studied have indicated that *in vitro* culture of ovarian tissue may influence on the follicular development through some alterations in the expression of genes involved in folliculogenesis (23, 26). Ovarian follicular development is a complex process (26) and the expression of different genes, such as proliferating cell nuclear antigen (*Pcna*), follicle-stimulating hormone receptor (*Fshr*), *P450*, and *Cyp17a1*, in this process are of importance, since changes in the expression of these genes may have some impacts on the process of follicle, oocyte or embryo development (23). PCNA is one of the essential regulators of cell cycle with a molecular weight of 36 kDa. Due to being a cofactor for DNA polymerase delta at S phase cycle, PCNA plays a significant role in repair of damaged DNA. PCNA is also considered as a very important marker in cell proliferation due to strong binding to cyclin D at S phase. A number of studies have reported the expression of this gene in the ovaries of different species during follicular development such as pigs (27), cows (28), baboons (29) and mice (30). Expression of *Pcna* increases in G and S phases, while it decreases in M phase. *Cyp17a1* is a critical factor that involves in folliculogenesis and biosynthesis of steroid hormones (31, 32). FSHR as an internal membrane cell surface receptor is expressed on the granulosa cells of secondary follicles and plays an essential role in the transmission of follicles to the antral stage (33, 34).

Given the importance of *Pcns*, *Fshr* and *Cyp17a1* genes in the development of ovarian follicles and due to lack of necessary information regarding their changes throughout the *in vitro* culture of vitrified ovaries, this study aimed to evaluate the expression of these genes in follicles after vitrification of mouse ovarian tissue using a two-step *in vitro* culture.

## Materials and Methods

All chemicals were obtained from Sigma Aldrich (Munich, Germany) except mentioned otherwise. In this experimental study, a group of neonate (n=30) and adult NMRI male mice (n=10) were kept in a cycle of 14 hours light and 10 hours dark, at 20-24ºC and 40-50% humidity. Approval for this study was obtained from the Ethics Committee for Animal Research of the Tarbiat Modares University (Ref No: 52/1637).

### Experimental design

After collection of neonate female mice ovaries (n=60), they were randomly divided into two studied groups as follows: vitrified and non-vitrified groups. Then some of the tissues in both groups were considered as non-cultured and subjected to morphological evaluation and the others were cultured. The whole ovaries were cultured on the inserts for 7 days. In vitrified group (n=15), the morphology of ovaries and the percentage of follicles were evaluated with histological studies.

In non-vitrified group (n=15), their preantral follicles were isolated and cultured in a threedimensional culture system for 12 days. Then, the rates of fertilization and embryo development were evaluated in collected metaphase (M) II oocytes. The expression of several genes related to folliculogenesis was evaluated using real time reverse transcription-polymerase chain reaction (RT-PCR) at the end of culture in collected follicles.

### Ovarian collection

To collect ovaries, neonate female mice were killed by cervical dislocation, removed their ovaries with surrounding tissues, dissected, and washed with alpha-Minimal Essential Medium (α-MEM, Gibco, UK) supplemented with 5% fetal bovine serum (FBS, Gibco, UK).

#### Vitrification and warming

The ovaries (n=25) were vitrified as previously described (35). Briefly, the ovaries were transferred into vitrification solution, EFS40, containing 40% (v/v) ethylene glycol, 30% (w/v) Ficoll 70% (w/v), and 1 M sucrose supplemented with 10% bovine serum albomin (BSA) for 5 minutes at room temperature. Then, they were loaded onto the Cryolock and plunged into liquid nitrogen for 1 week. Afterward, the Cryolock was sequentially placed into 1, 0.5 and 0.25 M sucrose solutions containing 10% BSA in α-MEM medium for 5 minutes at room temperature. After warming, the ovaries were incubated for 1 hour in α-MEM medium supplemented with 5% FBS under mineral oil at 37˚C in a humidified atmosphere of 5% CO_2_-95% air. Then some of these tissues were considered as non-cultured and the others as cultured groups.

#### Ovarian culture

The ovaries in both groups were cultured on Millicell-CM inserts (pore size of 0.4-μm, 30 mm diameter, Millipore Corp., Germany) in the 24- well plates at 37˚C and in a humidified atmosphere of 5% CO_2_-95% air for 1 week. The culture medium was α-MEM medium supplemented with 5% FBS, 1% insulin-transferrin-selenium medium (ITS, Gibco, UK), and 100 mIU/ml recombinant follicle stimulating hormone (rFSH or Gonal-F, Serono, Switzerland). Half of the medium in each well were replaced with fresh medium every other day during culture period.

#### Histological evaluation

At the first and last days of culture period (7 days), the ovaries in each group (n=5/each group) were fixed in Bouin’s solution, processed and embedded in paraffin wax. Then, they were sectioned serially into 5 μm-thick slices, mounted on a glass slide, and stained with hematoxylin and eosin (H&E). Every 5th section of each ovary was studied for counting the follicles. Classification of follicles was described previously (36). Briefly, primordial, primary and preantral follicles were considered as those had flattened granulosa cells surrounding the oocyte, one layer of cuboidal granulosa cells, and two or more layers of cuboidal granulosa cells, respectively. To prevent duplicate count, only follicles with one nucleus were counted. Follicles with an intact oocyte and organized granulosa cells were considered as normal follicles, whereas degenerated follicles contained piknotic oocyte nuclei, shrunken ooplasm, and/or disorganized granulosa cells.

#### Surface ovarian area

The images of follicles of both studied groups were prepared using an invert microscope with an attached DP11 digital camera (Olympus, Japan). Surface of ovary (n=5/each group) was calculated based on μm² using Digimizer software system (MedCalc Software, Belgium).

### Follicle isolation and assessment of ovarian follicle viability by trypan blue staining

Preantral follicles, 140-150 μm in diameter, were mechanically isolated from cultured ovaries in both groups (n=170/each group) by a 29 gauge needles under a stereomicroscope (Olympus, Japan). Selected preantral follicles had central oocyte, 2-3 layers of granulosa cells and a thin layer of theca cells. Some of isolated preantral follicles from the cultured ovaries in both groups (n=20/each group) were stained using trypan blue (0.4%). The survived follicles were those that were not stained, whereas the damaged follicles were those that were stained moderately blue. The other isolated follicles were cultured using a threedimensional culture system.

### The encapsulation and culture of isolated preantral follicles

The isolated preantral follicles (n=150/each group) were encapsulated in sodium alginate as defined previously. Briefly, the follicles were cultured in α-MEM medium supplemented with FBS, rFSH, ITS, penicillin, and streptomycin under mineral oil at 37˚C in a humidified atmosphere of 5% CO_2_-95% air for 12 day. The half of media was changed every other day during culture period

### Assessment of follicular diameter and development

The morphology of cultured follicles was assessed under an inverted microscope (Olympus, Japan) every 48 hours during culture period. The follicles with dark appearance were defined as degenerated ones. The follicle diameter (n=10/each group) was determined by precalibrated ocular micrometer (Olympus, Japan) using an inverted microscope (magnification: ×100) during culture period.

#### *In vitro* ovulation induction

After 12 days, ovulation induction was carried out by adding 1.5 IU/mL human chorionic gonadotropin hormone (hCG, Organon, Netherlands) to the culture media. Then, some of cultured follicles were collected and stored at -80˚C for molecular assessment (n=30/ each group), while in others cultured follicles (n=60/ each group), the released oocytes were scored as germinal vesicle, germinal vesicle breakdown and MII. The oocytes at MII stage were collected and subjected to insemination and assessment of the embryo development.

#### *In vitro* fertilization and embryo culture

In a three-dimensional culture system, after *in vitro* culture of isolated follicles, the collected oocytes at MII stage were inseminated with capacitated spermatozoa derived from cauda epididymis of 7-8- week old male NMRI mice (n=10) in the global medium (Life Global, USA) supplemented with 15 mg/ml BSA for 4-6 hours. Then, the oocytes were removed and placed into a 20-μl drop of global medium with 5 mg/ml BSA under mineral oil at 37˚C in a humidified atmosphere of 5% CO_2_-95% air. Fertilization and developmental rates of 2-cell, 4-cell, 8-cell, morula and hatching blastocyst embryos were evaluated daily for 120 hours.

### RNA extraction

Total RNA was extracted from collected follicles in vitrified and non-vitrified groups at the end of culture period (n=30 follicles/each group, 10 follicles for each replicate of experiments) using RNeasy Mini Kit (Qiagen, Germany). To eliminate any genomic DNA contamination, DNase treatment was performed after RNA extraction. Determination of RNA concentration was then performed using spectrophotometry (Shimadzu, Japan), and RNA samples were stored at -80˚C. The cDNA was synthesized by oligo (dT) primers and reverse transcriptase at 42˚C in 60 minutes using the cDNA Synthesis Kit (Thermo Scientific, EU), according to the manufacturer’s instructions, and stored at -20˚C.

### Real-time reverse transcriptase-polymerase chain reaction

Designed primers by GenBank (http://www.ncbi. nlm.nih.gov) and Allele ID software are shown in Table 1, and β-actin was used as housekeeping gene in this study. The Applied Biosystem Real Time PCR Cycler according to QuantiTect SYBR Green RT-PCR kit (Applied Biosystems, UK) was used. Amplification of reference and target genes was done in the same run for each sample. The protocol of real time RT-PCR was programmed as follows: the holding step at 95˚C for 5 minutes, cycling step at 95˚C for 15 seconds, 58˚C for 30 seconds, and 72˚C for 15 seconds, which was followed by a melt curve step at 95˚C for 15 seconds, 60˚C for 1 minute, and 95˚C for 15 seconds. The relative quantitation for target genes was determined using Pfaffl method. All experiments of real time RTPCR were replicated three times.

### Statistical analysis

All experiments were repeated at least three times. Values are given as mean ± standard error (SE). The data of follicular count, ovarian area and gene expression in the vitrified and nonvitrified groups were compared using paried t test. The percent of normal follicles at different developmental stages were analyzed by one-way ANOVA and tukey’s HSD was used post hoc tests. The statistical analysis was accomplished using the Statistical Package for the Social Sciences version 21 (SPSS, SPSS Inc., USA). A value of P<0.05 was considered as statistically significant.

## Results

### Morphology of ovaries

**Fig.1:**
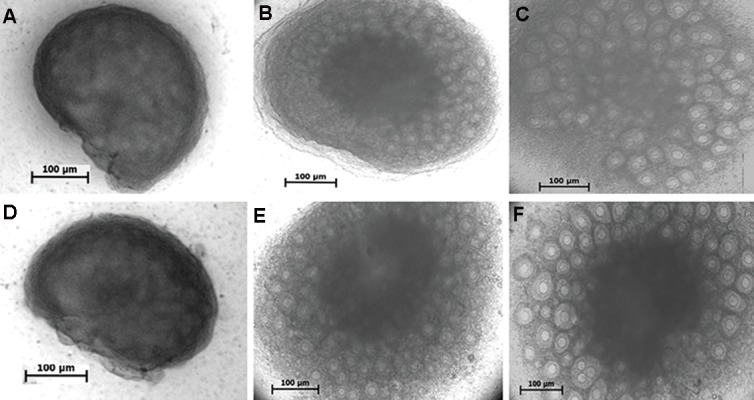
Photomicrographs of mouse ovaries under an inverted microscope during culture period are shown. The representative figures on days 0, 5, 7 in A-C. Non-vitrified and D-F. Vitrified groups are seen respectively.

**Fig.2:**
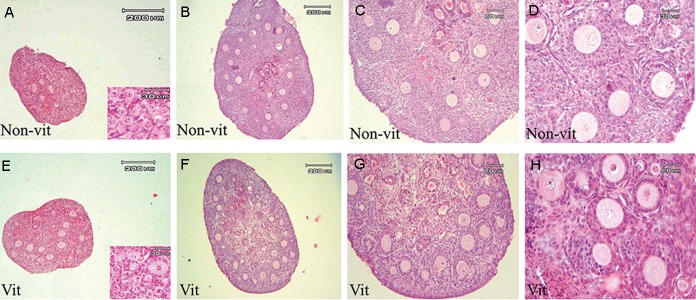
Photomicrographs of vitrified and non-vitrified whole mouse ovaries sections before and after 7-day culture using H&E staining. A. Non-cultured non-vitrified mouse ovary, B. Non-vitrified cultured ovary with low magnification, C, D. with high magnification, E. Vitrified non-cultured ovary, F. Vitrified-cultured ovary with low magnification, G, and H. with high magnification. It is noted that growing follicles are visible on day 7 of culture in non-vitrified and vitrified ovary, while degenerated follicles, especially in central areas of ovary, are demonstrated in both groups of study. It seems that the degenerated follicles in the vitrified samples are more than the non-vitrified group. H&E; Hematoxylin and eosin staining method.

The measurement values of phase contrast morphology of non-vitrified and vitrified ovaries on days 0, 5 and 7 of culture period are shown in Figure 1. The follicles were grown and the anterior surfaces of ovaries were swollen at the end of culture period in both groups. The dark areas in the central part of the cultured ovaries were detected in both groups; however, it was prominent in the vitrified sample. Figure 2 shows the morphology of vitrified and non-vitrified ovaries before and after culture period using H&E. Growing follicles with normal morphology were visible on day 7 of culture period in non-vitrified and vitrified ovaries. Moreover, degenerated follicles, especially in central area of ovaries, were detected in both groups. It seems that the degenerated follicles in the vitrified samples were more than the nonvitrified group.

**Table 1:** Designed primer sequences used for real-time reverse transcriptase- polymerase chain reaction (RT-PCR)


Gene	Primer sequence (5´-3´)	Accession numbers	PCR product size (bp)

*β-actin*	F: GGAAAAGAGCCTCAGGGCAT	NM_007393	64
	R: CTGCCTGACGGCCAGG		
*Pcna*	F: AGGAGGCGGTAACCATAG	NM-011045	76
	R: ACTCTACAACAAGGGGCACATC		
*Fshr*	F: CCAGGCTGAGTCGTAGCATC	NM-013523.3	79
	R: GGCGGCAAACCTCTGAACT		
*Cyp17a1*	F: CGCCGTCTGGGGAGAAACGGT	NM-007809.3	82
	R: CGTCAAAGACACCTGATGCCAAG		


### Percentage of normal follicles

The percentages of normal follicles in non-vitrified and vitrified groups before *in vitro* culture were 96.09 and 95.27%, while after one week *in vitro* culture, they were 73.95 and 73.42%, respectively. There was no significant difference regarding the normality rate of follicles between vitrified and non-vitrified groups, but it reduced in both cultured groups as compared with non-cultured samples (P<0.001). Among the normal follicles, the proportions of follicles in noncultured non-vitrified group at primordial, primary and preantral stages were 92.54, 5.31, and 2.14%, while these percentages in non-cultured vitrified group were 92.56, 5.32, and 2.12%, respectively (Table 2). In cultured non-vitrified group, after 7 days of culture, the percentages of follicles at primordial, primary and preantral stages were 65.48, 10.00, and 24.50%, while those of cultured vitrified group were 69.55, 11.18, and 18.40%, respectively. Many of follicles at primordial stage were grown to preantral stage during the culture period in both groups, indicating there was significant difference regarding percentage of preantral follicles before and after culture within each group (P<0.001). The proportion of preantral follicles in vitrified group was significantly lower than those of non-vitrified group on day 7 of culture (P<0.05).

**Table 2:** The percentages of follicles at different developmental stages in studied groups


Group	Total	Number of normal follicles (%)	Primordial folliclen (mean% ± SE)	Primary folliclen (mean% ± SE)	Preantral folliclen (mean % ± SE)

Non-cultured non-vitrified ovaries	3404	3271 (96.09)	3027 (92.58 ± 0.81)	174 (5.27 ± 0.65)	70 (2.1 ± 0.19)
Non-cultured vitrified ovaries	2960	2820 (95.27)	2610 (92.55 ± 1.61)	150 (5.31 ± 1.36)	60 (2.12 ± 0.32)
Cultured non-vitrified ovaries	1840	1359 (73.85)	890 (65.52 ± 1.16)^a^	136 (9.96 ± 0.48)^a^	333 (24.50 ± 1.07)^a^
Cultured vitrified ovaries	1554	1141 ( 73.42)	802 (69.56 ± 0.23)^b^	129 (11.19 ± 0.58)^b^	210 (18.41 ± 0.33)^b, c^


The percentage of follicles was calculated based on the normal follicles. ^a^; Significant difference with non-cultured non-vitrified ovaries (P<0.001), ^b^; Significant difference with non-cultured vitrified ovaries (P<0.001), ^c^; Significant difference with cultured non-vitrified ovaries (P<0.05), Real time PCR; Real time reverse transcription-polymerase chain reaction, and SE; Standard error.

### Area of ovaries

**Fig.3:**
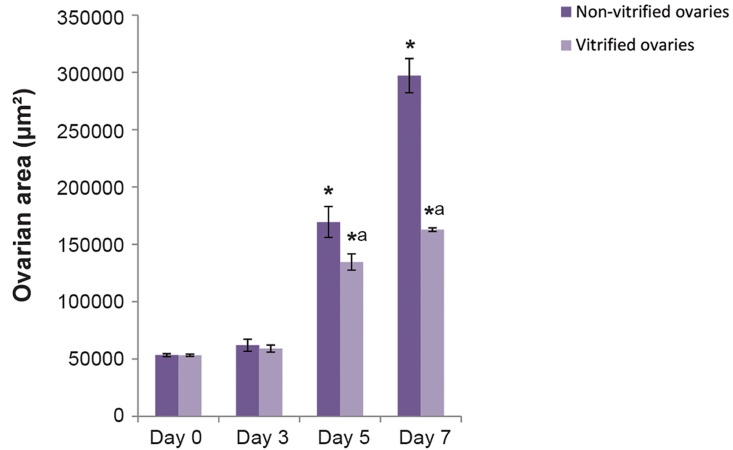
The mean area of mouse cultured vitrified and nonvitrified ovaries during a 7-day culture. a; There was a significant difference in this regard between two studied groups (P<0.002) and *; There were significant differences between days 7 and 5 with other days within each studied group (P<0.01)

The data of surface area analysis in cultured ovaries is presented in Figure 3. The mean areas in non-vitrified and vitrified cultured ovaries significantly increased from 53476.40 ± 568.97 and 53287.80 ± 410.44 μm² on day 0 to 297211.40 ± 6671.71 and 162468.20 ± 703.78 μm² on day 7 of culture, respectively (P<0.05). Surface areas of vitrified cultured ovaries were significantly lower than non-vitrified cultured ovaries on day 7 of culture (P<0.05).

### Survival rate of isolated preantral follicles

Survival rates of isolated preantral follicles from cultured non-vitrified and vitrified ovaries after culture period were 80.35 and 79.45%, respectively, suggesting that there was no significant difference in this regard between two treatment groups (P>0.05).

### Diameter of isolated preantral follicles

**Fig.4:**
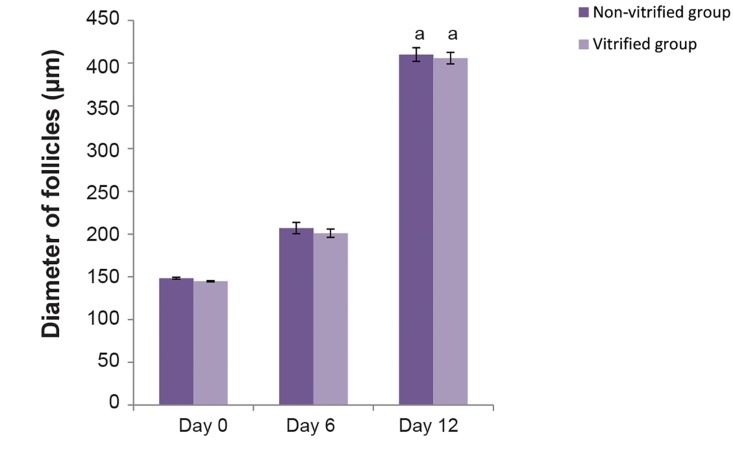
Diameter of cultured isolated preantral follicles in vitrified and non-vitrified groups. Values are given as mean ± standard deviation (SE). a; There was significant differences between days 12 with other days.

The mean diameters of cultured follicles in nonvitrified and vitrified ovaries groups significantly increased from 148.40 ± 1.14 and 144.80 ± 0.83 μm on day 0 to 410 ± 7.90 and 405.80 ± 6.72 μm on day 12, respectively (Fig .4, P<0.001), suggesting that there was no significant difference in this regard between two treatment groups during culture period (P>0.05).

### Developmental rate of follicles

The data related to development of follicles in both groups are summarized in Table 3. There was no significant difference regarding antrum formation between cultured non-vitrified and vitrified groups. The percentages of MII oocytes derived from cultured preantral follicles in nonvitrified and vitrified groups were 32.11 ± 2.39 and 30.00 ± 4.43, respectively. There were no significant differences in terms of the developmental rate of follicles and percent of matured oocytes reaching MII stage between two treatment groups (P>0.05).

### The rates of fertilization and embryo development

Fertilization rates of MII oocytes derived from cultured preantral follicles were 78.95 and 71.66% in cultured non-vitrified and vitrified groups, respectively (Table 4), indicating that there was no significant difference in this regard between two treatment groups (P>0.05).

### Gene expression analysis

**Fig.5:**
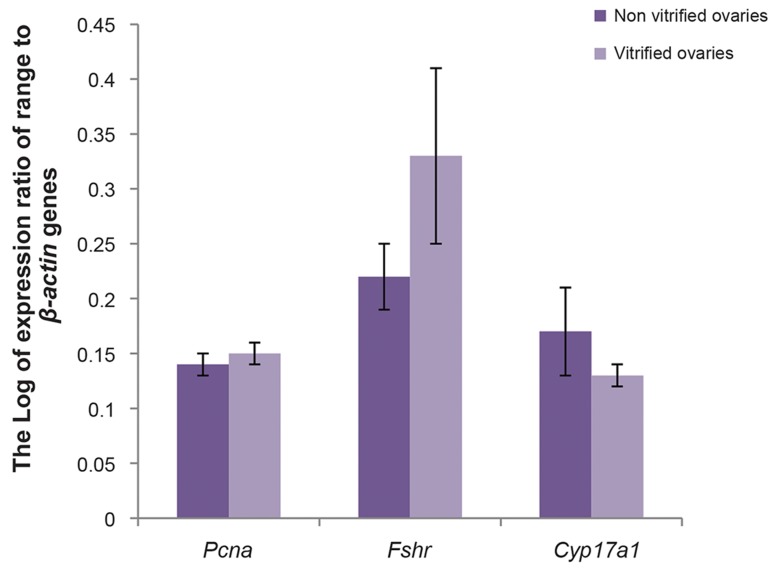
The mean expression ratios of *Pcna*, *Fshr* and *Cyp17a1* to a housekeeping gene (β-actin) by real time real time reverse transcription-polymerase chain reaction (RT-PCR) in cultured antral follicles on day 12 derived from whole ovarian culture. There was no significant difference in this regard between vitrified and non-vitrified groups (P>0.05).

The mean expression ratios of *Pcna*, *Fshr* and *Cyp17a1* to housekeeping gene in antral follicles derived from non- vitrified ovaries were 0.15 ± 0.01, 0.33 ± 0.14, and 0.13 ± 0.02, while these ratios in vitrified ovaries were 0.14 ± 0.01, 0.22 ± 0.05, and 0.17 ± 0.06, respectively (Fig .5). There were no significant differences in terms of the mean expression ratios of *Pcna*, *Fshr* and *Cyp17a1* to housekeeping gene between cultured vitrified and non-vitrified groups (P>0.05).

**Table 3:** The developmental and maturation rates of cultured preantral follicles


Group	Number of follicles	Survived (%)	Antrum formation (%)	Germinal vesicle (%)	MI (%)	MII (%)

Non-vitrified ovaries	102	76 (74.50)	45 (59.21)	22 (28.94)	31 (40.78)	23 (30.26)
Vitrified ovaries	136	100 (73.52)	58 (58)	29 (29)	41 (41)	30 (30)


The percentage was calculated based on the survived follicles. There was no significant difference between vitrified and non-vitrified groups (P>0.05). M; Metaphase.

**Table 4:** Fertilization and developmental rates of metaphase II oocytes in studied groups


Group	Number of MII	Number of fertilized (%)	Number of 2-cell (%)	Number of morula (%)	Number of hatched blastocyst (%)

Cultured non-vitrified ovaries	28	21 (78.95)	16 (77.33)	10 (45.33)	6 (29.33)
Cultured vitrified ovaries	25	18 (71.66)	14 (78.66)	8 (44.66)	5 (29.00)


## Discussion

Our findings showed that after one week in vitro culture of neonatal mouse ovarian tissue, there was a significant reduction in proportion of normal follicles in both vitrified and non-vitrified groups as compared to non-cultured tissues. However, this effect was more prominent in the vitrified group. It is suggested that *in vitro* culture condition is needed to be improved to enhance the follicular survival and development. Moreover, our results indicated that the vitrified sample was more sensitive to insufficient condition of the culture. Similarly, in our previous study, just after vitrification/warming, the integrity of tissue was well preserved; however, after one week in vitro culture, deleterious effects of vitrification on the follicular morphology and development were detected (37). In the present study, there were no significant differences between the follicular survival rate and antrum formation with the percentage of oocyte maturation in vitrified and non-vitrified groups at the end of two-stage culture of ovarian tissue, suggesting that vitrification had no negative impacts on follicular development at morphological level after *in vitro* culture of isolated follicles (steps two of *in vitro* culture). Moreover, our results demonstrated that a three-dimensional culture system provided an environment similar to human ovary in order to preserve the spherical shape of follicle, which is in agreement with a group of studies using a three-dimensional culture system for the growth of pre-antral follicles (38- 41). Abdi et al. (42) have recently showed that a three-dimensional culture system of isolated preantral follicles from cultured mouse ovarian tissue provided better conditions for survival and development of follicles as compared to the conventional two- dimensional culture system.

However, alteration in the gene expressions related to the oocyte maturation during culture period in both groups may influence subsequent embryo development. Results of the present study showed that there was no significant difference in gene expression of *Cyp17a1*, *Fshr* and *Pcna* in both vitrified and non-vitrified groups after a twostep culture that may confirm the morphological results in both groups at the molecular level.

*Pcna* as one of the factors in regulating development of ovarian follicles participates in cellular vital processes such as cell cycle control, cell survival, replication, repair and prevention of DNA damage; however, its exact function in meiosis, especially in formation of primordial follicles is still unknown. Xu et al. (30) have reported that reduced expression of *Pcna* caused a delay in transition of follicles from primordial to primary stage, while it reduced the proliferation of somatic cells. Choi et al. (43) have isolated and cultured preantral follicles of 12-day old mice after ovarian tissue vitrification. They have then reported that in the vitrification group, the expression of *Pcna* at 0 and 24 hours after culturing follicles significantly reduced in comparison with the control group. However, there was no significant difference between the two groups at 48 to 72 hours after culture. Their results have showed that proliferation of granulosa cell was delayed after vitrification, although it improved after 48 hours of culture period.

*Fshr* is expressed in granulosa cells of preantral follicles, and the reaction of this receptor with FSH hormone leads to follicle growth by proliferation and differentiation of granulosa cells and the formation of antrum (34). *Cyp17a1* has a key role in synthesis of steroid hormones. A group of studies have revealed that suppression of *Cyp17a1* decreased the levels of progesterone, androstenedione, testosterone and 17a-hydroxyprogesterone (17-OHP) (44- 46). Similarly, Fatehi et al. (23) cultured isolated preantral follicles from vitrified and non- vitrified 12- day old mouse ovarian tissues by a twodimensional culture system. Their results have indicated that the expression of some genes related to folliculogenesis (*Bmp15, Gdf9, BmprII, Alk6, Alk5, Has2* and *Ptgs2*) had not effects on vitrified isolated and cultured follicles as compared with the fresh control group.

In another study by Shams Mofarahe et al. (47), they have showed that there was no significant difference in terms of expression of genes related to folliculogenesis (*FIGLA, KIT Ligand, GDF9* and *FSHR*) between the fresh human ovarian tissue with vitrified- warmed samples. In contrast to our results, Asadzadeh et al. (24) have reported that expression of some genes related to development of follicles (*Timp-2* and *Mmp-2*) were altered after vitrification of *in vitro* culture of mouse ovarian tissues.

Isachenko et al. (18) evaluated human ovarian tissue after droplet vitrification method and 16 days culture in molecular level. Their findings have indicated that after both vitrification and culture, gene expression of *GAPDH* in ovarian tissue reduced, suggesting that this difference may be due to different methods used for vitrification or different culture periods. Our findings demonstrated that in spite of reduction in the percentage of preantral follicles and ovarian surface area after 7-day culture in the vitrified group in comparison with non-vitrified group, there was no significant difference between the two groups in expression of genes related to development of follicles, oocyte maturation, fertilization and embryo development rate. This result may be due to the damaged follicles during the vitrification process that were ignored from following 12-day culture. On the other hand, by isolation of follicles, the intact and healthy follicles or the ones with less injury during the vitrification process were selected after culture period. However, it is suggested that more genes are required to be evaluated after vitrification and culture of ovarian tissue. Furthermore, after embryo transfer, the implantation rate and *in vivo* embryo development are needed to be assessed.

## Conclusion

The results of this study showed that vitrification of ovarian tissue following *in vitro* culture of tissue had negative impact on survival and development of the follicles. However, no significant alteration was observed in development, gene expression and hormonal production of *in vitro* culture of preantal follicles derived from vitrified ovarian tissue as compared to the non-vitrified samples.

## References

[1] Wallace WH, Kelsey TW, Anderson RA (2016). Fertility preservation in pre-pubertal girls with cancer: the role of ovarian tissue cryopreservation. Fertil Steril.

[2] Suzuki N (2015). Ovarian tissue cryopreservation using vitrification and/or in vitro activated technology. Hum Reprod.

[3] Youm HW, Lee JR, Lee J, Jee BC, Suh CS, Kim SH (2014). Optimal vitrification protocol for mouse ovarian tissue cryopreservation: effect of cryoprotective agents and in vitro culture on vitrified-warmed ovarian tissue survival. Hum Reprod.

[4] Sadr SZ, Ebrahimi B, Shahhoseini M, Fatehi R, Favaedi R (2015). Mouse preantral follicle development in two-dimensional and three-dimensional culture systems after ovarian tissue vitrification. Eur J Obstet Gynecol Reprod Biol.

[5] Choi WJ, Seok JS, Choi IY, Park JK, Shin JK, Lee SA (2015). Expression of angiogenic factors in cryopreserved mouse ovaries after heterotopic autotransplantation. Obstet Gynecol Sci.

[6] Zeng YC, Tang HR, Zeng LP, Chen Y, Wang GP, Wu RF (2016). Assessment of the effect of different vitrification solutions on human ovarian tissue after short-term xenotransplantation onto the chick embryo chorioallantoic membrane. Mol Reprod Dev.

[7] Lee J, Kim EJ, Kong HS, Youm HW, Lee JR, Suh CS (2015). A combination of simvastatin and methylprednisolone improves the quality of vitrified-warmed ovarian tissue after auto-transplantation. Hum Reprod.

[8] Kagawa N, Silber S, Kuwayama M (2009). Successful vitrification of bovine and human ovarian tissue. Reprod Biomed Online.

[9] Tan X, Song E, Liu X, Liu G, Cheng H, Wan F (2012). Successful vitrification of mouse ovaries using less-concentrated cryoprotectants with Supercool X-1000 supplementation. In Vitro Cell Dev Biol Anim.

[10] Wang X, Catt S, Pangestu M, Temple-Smith P (2009). Live offspring from vitrified blastocysts derived from fresh and cryopreserved ovarian tissue grafts of adult mice. Reproduction.

[11] Milenkovic M, Diaz-Garcia C, Wallin A, Brännström M (2012). Viability and function of the cryopreserved whole rat ovary: comparison between slow-freezing and vitrification. Fertil Steril.

[12] Zhou XH, Wu YJ, Shi J, Xia YX, Zheng SS (2010). Cryopreservation of human ovarian tissue: comparison of novel direct cover vitrification and conventional vitrification. Cryobiology.

[13] Amorim CA, Van Langendonckt A, David A, Dolmans MM, Donnez J (2009). Survival of human pre-antral follicles after cryopreservation of ovarian tissue, follicular isolation and in vitro culture in a calcium alginate matrix. Hum Reprod.

[14] Telfer EE, Zelinski MB (2013). Ovarian follicle culture: advances and challenges for human and nonhuman primates. Fertil Steril.

[15] Araújo VR, Gastal MO, Figueiredo JR, Gastal EL (2014). In vitro culture of bovine preantral follicles: a review. Reprod Biol Endocrinol.

[16] Hasegawa A, Mochida N, Ogasawara T, Koyama K (2006). Pup birth from mouse oocytes in preantral follicles derived from vitrified and warmed ovaries followed by in vitro growth, in vitro maturation, and in vitro fertilization. Fertil Steril.

[17] Wang X, Catt S, Pangestu M, Temple-Smith P (2011). Successful in vitro culture of pre-antral follicles derived from vitrified murine ovarian tissue: oocyte maturation, fertilization, and live births. Reproduction.

[18] Isachenko V, Lapidus I, Isachenko E, Krivokharchenko A, Kreienberg R, Woriedh M (2009). Human ovarian tissue vitrification versus conventional freezing: morphological, endocrinological, and molecular biological evaluation. Reproduction.

[19] Chen JY, Li XX, Xu YK, Wu H, Zheng JJ, Yu XL (2014). Developmental competence and gene expression of immature oocytes following liquid helium vitrification in bovine. Cryobiology.

[20] Sales AD, Duarte AB, Santos RR, Alves KA, Lima LF, Rodrigues GQ (2016). Modulation of aquaporins 3 and 9 after exposure of ovine ovarian tissue to cryoprotectants followed by in vitro culture. Cell Tissue Res.

[21] Jafarabadi M, Abdollahi M, Salehnia M (2015). Assessment of vitrification outcome by xenotransplantation of ovarian cortex pieces in γ-irradiated mice: morphological and molecular analyses of apoptosis. J Assist Reprod Genet.

[22] Wang TR, Yan J, Lu CL, Xia X, Yin TL, Zhi X (2016). Human single follicle growth in vitro from cryopreserved ovarian tissue after slow freezing or vitrification. Hum Reprod.

[23] Fatehi R, Ebrahimi B, Shahhosseini M, Farrokhi A, Fathi R (2014). Effect of ovarian tissue vitrification method on mice preantral follicular development and gene expression. Theriogenology.

[24] Asadzadeh R, Khosravi S, Zavareh S, Ghorbanian MT, Paylakhi SH, Mohebbi SR (2016). Vitrification affects the expression of matrix metalloproteinases and their tissue inhibitors of mouse ovarian tissue. Int J Reprod Biomed (Yazd).

[25] Sampaio da Silva AM, Bruno JB, de Lima LF, Ribeiro de Sá NA, Lunardi FO, Ferreira AC (2016). Connexin 37 and 43 gene and protein expression and developmental competence of isolated ovine secondary follicles cultured in vitro after vitrification of ovarian tissue. Theriogenology.

[26] Parrish EM, Siletz A, Xu M, Woodruff TK, Shea LD (2011). Gene expression in mouse ovarian follicle development in vivo versus an ex vivo alginate culture system. Reproduction.

[27] Tománek M, Chronowska E (2006). Immunohistochemical localization of proliferating cell nuclear antigen (PCNA) in the pig ovary. Folia Histochem Cytobiol.

[28] Isobe N, Yoshimura Y (2000). Immunocytochemical study of cell proliferation in the cystic ovarian follicles in cows. Theriogenology.

[29] Wandji SA, Srsen V, Nathanielsz PW, Eppig JJ, Fortune JE (1997). Initiation of growth of baboon primordial follicles in vitro. Hum Reprod.

[30] Xu B, Hua J, Zhang Y, Jiang X, Zhang H, Ma T (2011). Proliferating cell nuclear antigen (PCNA) regulates primordial follicle assembly by promoting apoptosis of oocytes in fetal and neonatal mouse ovaries. PLoS One.

[31] Taniguchi F, Couse JF, Rodriguez KF, Emmen JM, Poirier D, Korach KS (2007). Estrogen receptor-alpha mediates an intraovarian negative feedback loop on thecal cell steroidogenesis via modulation of Cyp17a1 (cytochrome P450, steroid 17alpha-hydroxylase/17,20 lyase) expression. FASEB J.

[32] Erickson GF, Magoffin DA, Dyer CA, Hofeditz C (1985). The ovarian androgen producing cells: a review of structure/function relationships. Endocr Rev.

[33] Zheng W, Magid MS, Kramer EE, Chen YT (1996). Follicle-stimulating hormone receptor is expressed in human ovarian surface epithelium and fallopian tube. Am J Pathol.

[34] Sullivan RR, Faris BR, Eborn D, Grieger DM, Cino-Ozuna AG, Rozell TG (2013). Follicular expression of follicle stimulating hormone receptor variants in the ewe. Reprod Biol Endocrinol.

[35] Salehnia M, Abbasian Moghadam E, Rezazadeh Velojerdi M (2002). Ultrastructure of follicles after vitrification of mouse ovarian tissue. Fertil Steril.

[36] Yang MY, Fortune JE (2008). The capacity of primordial follicles in fetal bovine ovaries to initiate growth in vitro develops during mid-gestation and is associated with meiotic arrest of oocytes. Biol Reprod.

[37] Abdi S, Salehnia M, Hosseinkhani S (2015). Kit ligand decreases the incidence of apoptosis in cultured vitrified whole mouse ovaries. Reprod Biomed Online.

[38] Jin SY, Lei L, Shikanov A, Shea LD, Woodruff TK (2010). A novel two-step strategy for in vitro culture of early-stage ovarian follicles in the mouse. Fertil Steril.

[39] Ahn JI, Kim GA, Kwon HS, Ahn JY, Hubbell JA, Song YS (2015). Culture of preantral follicles in poly(ethylene) glycol-based, three-dimensional hydrogel: a relationship between swelling ratio and follicular developments. J Tissue Eng Regen Med.

[40] Desai N, Abdelhafez F, Calabro A, Falcone T (2012). Three dimensional culture of fresh and vitrified mouse pre-antral follicles in a hyaluronan-based hydrogel: a preliminary investigation of a novel biomaterial for in vitro follicle maturation. Reprod Biol Endocrinol.

[41] Asgari F, Valojerdi MR, Ebrahimi B, Fatehi R (2015). Three dimensional in vitro culture of preantral follicles following slow-freezing and vitrification of mouse ovarian tissue. Cryobiology.

[42] Abdi S, Salehnia M, Hosseinkhani S (2016). Quality of oocytes derived from vitrified ovarian follicles cultured in two- and three-dimensional culture system in the presence and absence of kit ligand. Biopreserv Biobank.

[43] Choi J, Lee B, Lee E, Yoon BK, Bae D, Choi D (2008). Cryopreservation of ovarian tissues temporarily suppresses the proliferation of granulosa cells in mouse preantral follicles. Cryobiology.

[44] Pelletier G, Li S, Luu-The V, Tremblay Y, Bélanger A, Labrie F (2001). Immunoelectron microscopic localization of three key steroidogenic enzymes (cytochrome P450(scc), 3 beta-hydroxysteroid dehydrogenase and cytochrome P450(c17)) in rat adrenal cortex and gonads. J Endocrinol.

[45] Corbin CJ, Moran FM, Vidal JD, Ford JJ, Wise T, Mapes SM (2003). Biochemical assessment of limits to estrogen synthesis in porcine follicles. Biol Reprod.

[46] Wen X, Li D, Tozer AJ, Docherty SM, Iles RK (2010). Estradiol, progesterone, testosterone profiles in human follicular fluid and cultured granulosa cells from luteinized preovulatory follicles. Reprod Biol Endocrinol.

[47] Shams Mofarahe Z, Ghaffari Novin M, Jafarabadi M, Salehnia M, Noroozian M, Ghorbanmehr N (2015). Effect of human ovarian tissue vitrification/warming on the expression of genes related to folliculogenesis. Iran Biomed J.

